# BDNF Plasma Levels and BDNF Exon IV Promoter Methylation as Predictors for Antidepressant Treatment Response

**DOI:** 10.3389/fpsyt.2018.00511

**Published:** 2018-10-26

**Authors:** Klaus Lieb, Nadine Dreimüller, Stefanie Wagner, Konrad Schlicht, Tanja Falter, Alexandra Neyazi, Linda Müller-Engling, Stefan Bleich, André Tadić, Helge Frieling

**Affiliations:** ^1^Department of Psychiatry and Psychotherapy, University Medical Centre, Mainz, Germany; ^2^Institute of Clinical Chemistry and Laboratory Medicine, University Medical Centre, Mainz, Germany; ^3^Molecular Neuroscience Laboratory, Department of Psychiatry, Social Psychiatry and Psychotherapy, Hannover Medical School (MHH), Hanover, Germany

**Keywords:** BDNF-promoter methylation, BDNF plasma level, MDD, early improvement, response prediction

## Abstract

Major problems of current antidepressant pharmacotherapy are insufficient response rates and difficulties in response prediction. We recently provided preliminary evidence in a small study that patients with major depressive disorder (MDD) with a hypomethylation of the CpG-87 site of the promoter IV region of the brain-derived neurotrophic factor (BDNF) gene are less likely to benefit from antidepressants. Here, we aimed at replicating this finding in a secondary analysis of 561 MDD patients (mean age 40.0 ± 11.9 years, 56% female) included into the Early Medication Change study (EMC). We measured BDNF exon IV promoter and p11 gene methylation at Baseline (BL) as well as BDNF-plasma-levels (pBDNF) at BL and day 14 and related them to treatment outcome. Although we were not able to replicate the predictor function of hypomethylation of the BDNF exon IV promoter, a subgroup of patients with severe depression (Hamilton Depression Rating Scale [HAMD-17] ≥ 25) (*n* = 199) and hypermethylation at CpG-87 of the BDNF exon IV promoter had significantly higher remission rates than patients without a methylation (*p* = 0.032). We also found that 421 (75%) of 561 patients showed an early improvement (≥ 20% HAMD-17 reduction after 2 weeks), which was associated with a 4.24-fold increased likelihood to remit at study end compared to the 140 patients without early improvement. However, specificity of response prediction of early improvement was low (34%) and false positive rate high (66%). The combination of early improvement with a pBDNF increase between BL and day 14, however, increased the specificity of response prediction from 34 to 76%, and the combination with methylation of the CpG-87 site of the BDNF exon IV promoter from 34 to 62%. Thus, the combined markers reduced false positives rates from 66 to 24% and 38%, respectively. Methylation at other sites or p11 promoter methylation failed to increase specificity of early improvement prediction. In sum, the results add to previous findings that BDNF, BDNF promoter methylation and the combination of clinical and biological markers may be interesting candidates for therapy response prediction which has to be confirmed in further studies.

**Clinical Trial Registration:** https://clinicaltrials.gov/ct2/show/NCT00974155, identifier: NCT00974155

## Introduction

Antidepressant pharmacotherapy with monoaminergic drugs leads to insufficient responses in up to two-third of patients with major depressive disorder (MDD) and this is a key problem in the treatment of patients since therapy failure is normally determined only after several weeks of unsuccessful treatment (1–4). This long period until determination of treatment response asks for early clinical or biological markers to predict later treatment response in patients with MDD.

In recent years, evidence has accumulated that a combination of clinical markers with biomarkers such as blood immune markers, theta-cordance, executive test performance may improve treatment prediction (5–7). An especially promising candidate for a biological marker is brain-derived neurotrophic factor (BDNF) (7–11). Several lines of evidence have linked BDNF with both the pathophysiology of depression and the mode of action of antidepressants (12–14). Studies have shown in rodents that antidepressants including ketamine and electroconvulsive therapy (ECT) increased BDNF levels in cortex and hippocampus (15) and that BDNF protein infusion in hippocampal areas led to antidepressant-like effects (12). Furthermore, animal models of depression showed that antidepressant-like responses were dependent on BDNF/TrkB signaling (12, 16, 17). An important role of BDNF for antidepressant response was also shown in knockout studies or by pharmaceutical inhibition of BDNF which both prevented the efficacy of a variety of different therapeutic antidepressant approaches including non-pharmacological treatments such as sleep deprivation and ECT (14).

Clinical improvement and antidepressant therapy are not only related to BDNF in the brain but also to an increase of BDNF in human blood (18). Accordingly, peripheral BDNF levels can serve as a biomarker for the successful treatment of depression (10) and are relevant markers for the state of MDD (19). In a recent meta-analysis, a significant interaction between serum/plasma BDNF and antidepressant therapy was found, showing an increase of peripheral BDNF in patients treated with antidepressants (20).

Other studies demonstrated that antidepressant treatment increased central BDNF levels in animals (12) as well as peripheral BDNF levels in humans (10, 20–22). However, these finding are inconsistent, at least in humans, as other studies have shown decreases of peripheral BDNF levels during the course of treatment as well (23), no change at all (24) or differences between antidepressants (25). An especially interesting biological marker for therapy response prediction may be BDNF exon IV promoter methylation which has gained high interest in recent years, as it was shown that especially this promoter site controls BDNF expression (26). Also P11 (also known as S100A10) is an interesting candidate. It is a member of the S100 gene family that acts as an adaptor protein and is critically involved in amplification of serotonergic signaling and the regulation of gene transcription (27). In a mouse model of MDD as well as in MDD patients, P11 is down regulated and levels rise by administration of SSRIs or electroconvulsive therapy. Antidepressant effects of BDNF and ketamine have been shown to be mediated by P11 (28–30) and BDNF induces p11 by signaling using the ERK pathway (31).

In previous studies, we have used several BDNF related biomarkers to predict treatment outcome in MDD patients. We found that non-remission by antidepressant pharmacotherapy was predicted by hypomethylation of a specific CpG site (m87) in the BDNF exon IV promoter (11), and we obtained similar findings for BDNF exon I methylation and response to ECT treatment (32). In additional small studies, we described that non-response and non-remission were predicted by a non-increase of BDNF in serum (33) or plasma (7) during the first week of antidepressant treatment. This suggests that changes in peripheral BDNF during the early course of treatment may constitute or reflect a necessary prerequisite for a later treatment response.

A promising clinical marker for treatment response prediction is an early improvement of depressive symptoms, mostly defined as a ≥20% reduction in sum scores of depressive rating scales between baseline and day 14 (34, 35). In a recent meta-analysis including data from 14,799 patients, we showed that patients with an early improvement of depressive symptomatology after 2 weeks of antidepressant treatment had an 8-/6.5-fold increased likelihood to become responder/remitter at treatment end as compared to non-improver (36). However, although early improvement shows a high sensitivity, it has only a low specificity (true negative rate) meaning that many early improvers become later non-responders (37%) or non-remitters (67%) (36). The low specificity of the prediction of early improvement, therefore, asks for additional markers (e.g., biological markers), which could be combined with the early improvement marker to improve the specificity of prediction of treatment outcome.

In the current study, we used a large sample of patients with MDD (n = 561) to replicate our previous findings of a predictive role of BDNF exon IV promoter hypomethylation (11) and early peripheral BDNF changes (7, 33) for remission of MDD. As it is well-established that treatment outcome is depending on the degree of depression severity with antidepressants being particularly efficient in patients with severe to very severe depression (37), we repeated all analyses for the subgroup of patients with at least a severe symptomatology (*N* = 199). Furthermore, we used our sample to analyze whether the combined marker of early improvement and BDNF-related markers increases the specificity of response prediction, as previous studies showed that adding of biological markers to early improvement can improve response prediction (5). Studies investigating the predictive power of combined markers found encouraging results (38). We were especially interested to see whether the following markers predict later remission or increase the specificity of therapy response prediction by early symptomatic improvement: (i) methylation status at BDNF promoter exon IV CpG-87 at baseline, as well as (ii) promoter methylation status of the multifunctional protein p11 (S100Δ10), (iii) BDNF plasma levels (pBDNF) at baseline, (iv) change of plasma BDNF levels from baseline to week 2.

## Materials and methods

### Patients

This study is a secondary scientific investigation in 561 patients with MDD who had participated between 2009 and 2014 in the “Randomized clinical trial comparing an early medication change (EMC) strategy with treatment as usual (TAU) in patients with Major Depressive Disorders (MDD)—The EMC Trial (ClinicalTrials.gov identifier n°: NCT00974155)” and who had agreed to biomarker sampling. The EMC trial was carried out in accordance with the recommendations of consort guidelines, ethics committee at the Landesärztekammer Rheinland-Pfalz. The protocol was approved by the ethics committee at the Landesärztekammer Rheinland-Pfalz, Germany. All subjects gave written informed consent in accordance with the Declaration of Helsinki. Clinical and demographical data of the 561 patients are given in Table 1. None of these subjects had participated in our previous pilot studies (7, 11, 39). Table 1 also gives demographic data for the 199 patients who suffered from a severe MDD (defined as Hamilton Rating Scale for Depression; HAMD-17 ≥ 25) and who were analyzed separately as a subgroup. Details of the protocol have been described previously (39–42). In short, the EMC study was a multi-center, randomized, observer-blinded, controlled clinical trial investigating whether non-improver after 14 days of an antidepressant treatment with escitalopram are more likely to remit (HAMD-17 ≤ 7) after 8 weeks of treatment with an early medication change (EMC: immediate change to venlafaxine followed by an augmentation with lithium after non-response at day 28) compared to patients treated according to current guideline recommendations (TAU: continuing escitalopram for 2 weeks followed by venlafaxine in the case of non-response). Key inclusion criteria of the EMC trial were: (1) Major Depressive Disorder (MDD), first episode or recurrent, according to DSM-IV; (2) a HAMD-17 score of ≥ 18 points at screening; (3) age 18–65 and ≤ 60 years at the time of the first depressive episode. Minimal exclusion criteria were used to maximize generalizability. Patients with (1) a primary diagnosis of bipolar, psychotic, obsessive-compulsive, eating disorder or substance dependence (if it required inpatient detoxification); (2) female patients who were pregnant or breast-feeding; (3) patients with general medical conditions contraindicating the use of any protocol medication, or (4) a clear history of non-response or intolerance in the current MDD episode to any protocol antidepressant were excluded from the study.

**Table 1:** Clinical and demographic characteristics of patients at treatment initiation.

**Characteristic**	**All patients (*n* = 561)**	**Severe depressed patients (*n* = 199)**
**SOCIODEMOGRAPHIC DOMAIN**		
Age—years (± SD)	40.0 ± 11.9	41.2 ± 11.2
Sex		
Female—*n* (%)	315 (56%)	118 (59%)
Male—*n* (%)	246 (44%)	81 (41%)
Ethnicity		
Caucasian—*n* (%)	546 (97%)	191 (96%)
**CLINICAL DOMAIN**		
Age at onset—years (± SD)	32.2 ± 12.2	32.2 ± 11.9
Course of depression		
1st episode—*n* (%)	192 (34%)	65 (33%)
recurrent—*n* (%)	369 (66%)	134 (67%)
Previous episodes—*n* (± SD)	4.1 ± 5.6	4.1 ± 6.0
Duration of index major depressive episode—weeks (± SD)	34.0 ± 59.4	31.8 ± 38.6
HAMD-17 sum score at baseline	24.1 ± 4.1	28.0 ± 2.8

*HAMD, Hamilton Depression Rating Scale; SD, standard deviation; n, number*.

### Study procedures

At screening visit, the diagnosis was verified by a structured interview: M.I.N.I. International Neuropsychiatric Interview (43), according to DSM-IV, Axis II Disorders by the Structured Clinical Interview for DSM-IV Axis II Disorders (SCID-II) (44). Demographic parameters (age, sex, ethnicity) and psychiatric history (number of preceding depressive episodes, length of index episode, age at onset, clinical course) were assessed relying on patients' self-reports (41, 42). The severity of depressive symptomatology was assessed weekly by the Hamilton Depression Rating Scale: HAMD-17 (45) by blinded and specially trained raters (46). Blood samples were also obtained weekly as previously described (39). All blood samples were taken between 08:00 and 12:00 h in the morning and placed within a time frame of 30 min on ice after collection. In this study, BDNF plasma levels (pBDNF) were measured at baseline, week 2, and methylation status of BDNF exon IV promoter and p11 promoter at baseline.

### BDNF exon IV promoter and p11 promoter methylation analysis

Genomic DNA was isolated from 200 μL frozen human venous blood using the NucleoMag® Blood 200 μL Kit (Macherey & Nagel, Dueren) on a Biomek® NXP Laboratory Automation Workstation (Beckman Coulter, Brea, CA). Afterwards, 500 ng of genomic DNA were modified by sodium-bisulfite using the EpiTect® 96 Bisulfite Kit (QIAGEN).

DNA was amplified through (semi-) nested touch-down PCR. Primer sets for amplification of BDNF and p11 promoter region (Metabion GmbH, Steinkirchen, Germany) are listed in Supplementary Table 1. All PCRs were performed on a C1000^TM^ Thermal Cycler (BioRad, Hercules CA, USA). Subsequently 10 μl of each PCR product were visualized on a standard 2.0% agarose gel and the remaining 40 μl were purified using Agencourt® AMPure®XP magnetic beads on a BioMek NX^P^ liquid handling system (Beckman Coulter) and subsequently sequenced using the reverse primer via by BigDye® Terminator v3.1 Cycle Sequencing Kit (Applied Biosystems, Foster City, CA, USA) and an Applied Biosystems 3,500 × l Genetic Analyzer (Applied Biosystems). Sequences and electropherograms were analyzed via the specialized Epigenetic Sequencing Methylation (ESME) analysis software (47) and the percentage methylation of each CpG site within the amplified region was estimated by the ratio between peak values of Cytosine (C) and Thymine (T) (C/T).

### Measurement of BDNF plasma levels

Whole blood was obtained in a lithium-heparin tube from baseline to day 56 in weekly intervals. After a maximum time of 30 min, whole blood was centrifuged at 1,000 × g at 4°C to separate plasma. Plasma was then pipetted into Eppendorf tubes; these were centrifuged at 10,000 × g and at 4°C. Plasma was pipetted in small Eppendorf tubes again, then kept at −80°C. Plasma BDNF concentration was assessed with a commercially available kit (Quantikine ELISA, Human free BDNF Immunoassay, R&D Systems Europe) according to the instructions of the manufacturer. All plasma samples were double-analyzed with the Human free BDNF Immunoassay; an internal control sample was continuously measured with each microtiter plate. Inter-assay coefficient of variation in our sample was 11.2% and intra-assay coefficient of variation was 6.3%.

### Predictors and outcome parameter

As clinical predictor for remission (defined as a HAMD-17 sum score < 7 at endpoint) we used the occurrence of early improvement, defined as a ≥20% reduction in sum score of the HAMD-17 between baseline and day 14. Dropouts before day 28 were counted as non-remitters; dropouts after day 28 were counted as remitters or non-remitters according to the last HAMD-17 sum score. We furthermore assessed three sets of molecular markers and used them as predictors of remission at endpoint and combined them with the clinical marker early improvement to enhance specificity of response prediction: BDNF exon IV promoter methylation (at CpG03m87, 01m147, 02m111, 04m66, 05m58, 06m39, 07m35, 08m24, 10p18) and p11 methylation (at position 38, 44, 78, 112, 114, 128, 207, 211, 216, 244, 254, 256, 260, 314) at baseline, as well as BDNF plasma levels (at baseline and after 2 weeks) and change of BDNF plasma levels (between baseline and week 2). To assess the value of therapy response prediction by a combination of early improvement and BDNF markers, it was important to measure the biomarkers exactly at the same time, i.e., at baseline and after 2 weeks.

### Statistical analysis

Differences in the number of early improver/non-improver, and remitter/non-remitter with or without methylation at BDNF exon IV promoter were analyzed by Chi^2^-tests. Correlation analyses were used to assess the effect of potential covariates like age, gender or education on pBDNF, BDNF exon IV promoter or P11 methylation. Significant covariates were included in all further analyses. Regarding BDNF exon IV promoter methylation status, the analyses focused on the dichotomous markers, due to our research question of therapy response “with” or “without” methylation. The methylation status (methylated/not methylated) could only be analyzed at 5 sites (CpG03m87, CpG04m66, CpG07m35, CpG08m24, CpG10p18), since the number of patients without a methylation was too small at the other CpG sites (*N* ≤ 8).

Differences in mean P11 promoter methylation levels at baseline and mean pBDNF levels (at baseline, change from BL to day 14) were analyzed by *t*-tests for independent variables. Differences in the change of BDNF from BL to day 14 between patients with or without methylation were analyzed by *t*-test for independent variables.

Logistic regression analyses with remission as outcome and the molecular markers (BDNF exon IV promoter methylation, P11 promoter methylation, pBDNF) as predictor were used to investigate the association between molecular markers and treatment outcome.

To assess the predictive value of early improvement and the molecular markers on treatment outcome, sensitivity, specificity, positive (PPV) and negative predictive values (NPV) as well as the Odd's ratios (OR) (95%-Confidence interval) were calculated. A sensitivity or specificity lower than 45% is estimated as low, a value between 45 and 70% as moderate, 71–90% as high and a sensitivity and specificity >90% as very high (48).

To further assess a possible predicting role of a combined molecular and clinical marker of early symptomatic improvement, we analyzed a combined marker consisting of the molecular markers plus an early improvement of the depressive symptomatology after 2 weeks of treatment for prediction of remission at study end. For the molecular marker, BDNF methylation was dichotomized (methylated/not methylated). The combined marker consists of four subgroups, i.e., early improver with methylation, early improver without methylation, early non-improver with methylation and early non-improver without methylation. The different components were weighed equally and improvement was included in a single calculation. Regarding pBDNF and p11 methylation levels at baseline, a median split was used to dichotomize the markers (below median, above median). Regarding a pBDNF change from baseline to day 14, patients with a pBDNF increase were compared to those with a decrease. Differences between patients with a pBDNF increase or decrease in the number of early improver/non-improver or remitter/non-remitter were calculated by Chi^2^-tests. Again, sensitivity, specificity, positive (PPV) and negative predictive values (NPV) as well as the Odd's ratios (95%-CI) were calculated. All analyses report the whole sample first, then those with severe MDD. All analyses were done using SPSS 23.0. Significance was set at *p* ≤ 0.050.

## Results

### BDNF markers in patients according to clinical course of treatment

#### BDNF exon IV promoter methylation

**Figure 1:**
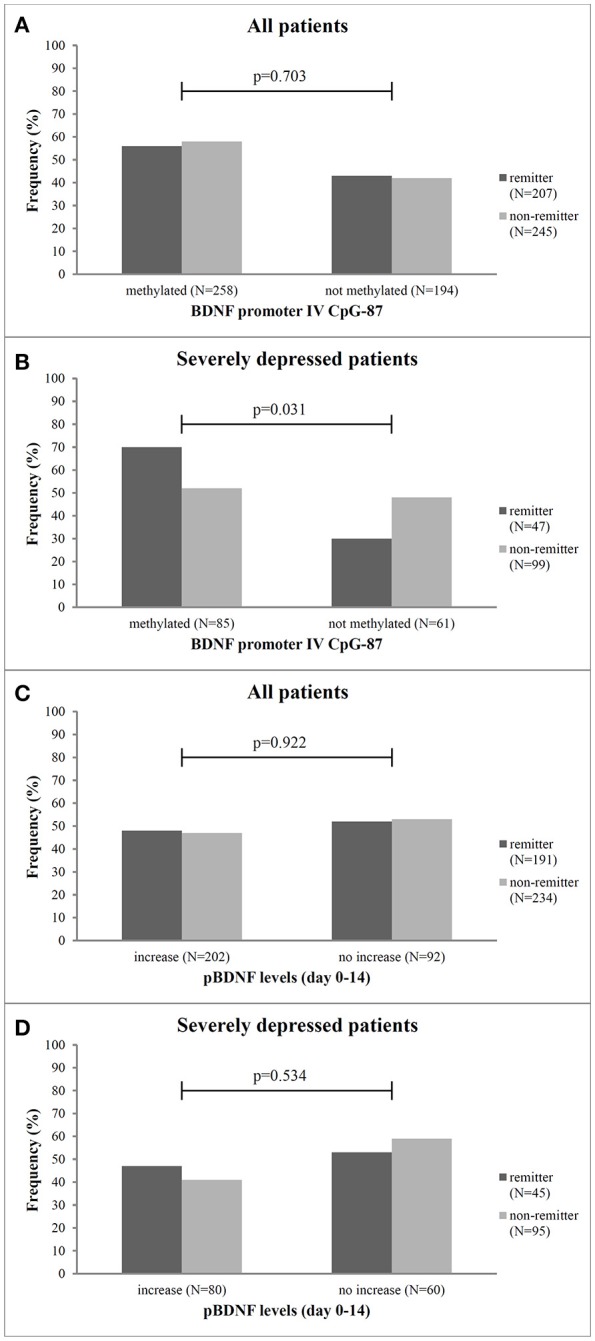
Frequency of remitter and non-remitter in relation to methylation at CpG-87 and change of pBDNF levels between baseline and day 14 for all patients (**A,C**, respectively) and the subgroup of severely depressed patients (**B,D**, respectively). N, number, p, Chi^2^-test.

No gender differences or effect of age in methylation status were found in our sample (*p* = 0.287). Patient groups with or without a methylation at BDNF promoter exon IV did not differ in the frequency of remitter (*p* = 0.703) after 8 weeks of antidepressant treatment at any of the investigated sites (Figure 1A). The logistic regression analysis with remission as dependent variable and the methylation status (dichotomic) at the investigated sites as criterion revealed no association between methylation status and treatment outcome for any site (*p* = 0.084). As shown in Table 2A, the methylation status at BDNF promoter exon IV predicted later remission with low to moderate sensitivity and specificity.

**Table 2:** Sensitivity and specificity of early improvement and its combination with BDNF markers for prediction of remission in all patients with MDD **(A)** and in the subgroup of severely depressed patients **(B)**.

**(A)**
**ALL patients**	**Sensitivity (95%-CI)**	**Specificity (95%-CI)**	**PPV (95%-CI)**	**NPV (95%-CI)**	**Odd's ratio (95%-CI)**
**OUTCOME: REMISSION AT ENDPOINT**					
**BDNF promoter exon IV methylation**					
CpG-87	56 (52–60)	42 (38–46)	45 (41–49)	53 (49–57)	0.92 (0–2)
CpG-66	77 (73–80)	22 (0–2)	45 (41–49)	53 (49–57)	0.94 (0–2)
CpG-35	72 (68–76)	23 (20–27)	44 (40–48)	49 (45–53)	0.95 (0–2)
CpG-24	52 (48–56)	40 (36–44)	42 (38–46)	50 (46–54)	0.73 (0–2)
CpG-18	38 (34–42)	57 (53–61)	43 (39–47)	52 (48–56)	0.82 (0–2)
**Combined marker early improvement plus BDNF promoter exon IV methylation**					
CpG-87	50 (42–58)	62 (54–70)	54 (46–62)	58 (50–66)	1.62 (0–4)
CpG-66	97 (95–98)	12 (9–15)	52 (48–56)	78 (74–81)	3.87 (2–6)
CpG-35	71 (67–75)	25 (22–29)	50 (46–54)	44 (40–48)	0.80 (0–2)
CpG-24	46 (42–50)	51 (47–55)	47 (43–51)	50 (46–54)	0.88 (0–2)
CpG-18	37 (33–41)	57 (53–61)	49 (45–53)	46 (42–50)	0.79 (0–2)
**Plasma BDNF**					
Baseline	47 (43–51)	56 (52–60)	48(44–52)	55 (51–59)	1.12 (0–2)
Δ BL - day 14	48 (44–52)	53 (49–57)	46 (42–50)	56 (52–60)	1.06 (0–2)
**Combined marker early improvement plus plasma BDNF**					
Baseline	86 (83–89)	36 (32–40)	55 (51–59)	75 (71–79)	3.52 (2–5)
Δ BL - day 14	55 (51–59)	76 (72–80)	88 (85–91)	33 (29–37)	3.78 (2–6)
**Early Improvement baseline—day 14**					
	89 (86–92)	34 (30–38)	48 (44–52)	82 (79–85)	4.24 (3–6)
**(B)**
**Severe depressed**	**Sensitivity (95%-CI)**	**Specificity (95%-CI)**	**PPV (95%-CI)**	**NPV (95%-CI)**	**Odd's ratio (95%-CI)**
**OUTCOME: REMISSION AT ENDPOINT**					
**BDNF promoter exon IV methylation**					
CpG-87	71(64–78)	74 (67–81)	40 (32–48)	77 (70–84)	2.96 (0–6)
CpG-66	33 (25–41)	30 (23–38)	29 (22–36)	34 (26–42)	0.21 (0–1)
CpG-35	67 (59–75)	24 (17–31)	29 (22–36)	61 (53–69)	0.65 (0–2)
CpG-24	33(25–41)	32 (24–40)	31 (38–46)	34 (23–39)	0.24 (0–1)
CpG-18	40 (32–48)	51 (43–59)	27 (20–34)	64 (56–72)	0.69 (0–2)
**Combined marker early improvement plus BDNF promoter exon IV methylation**					
CpG-87	60 (52–68)	63 (55–71)	45 (37–53)	77 (69–84)	2.63 (0–5)
CpG-66	80 (73–87)	40 (32–48)	38 (30–46)	81 (75–87)	2.64 (0–5)
CpG-35	57 (59–65)	41 (37–47)	32 (24–40)	67 (59–74	0.95 (0–3)
CpG-24	43 (35–51)	53 (45–61)	30 (26–38)	65 (57–73)	0.82 (0–2)
CpG-18	32 (24–40	64 (56–71)	29 (25–37)	66 (58–74)	0.82 (0–2)
**Plasma BDNF**					
Baseline	55 (47–63)	61 (53–69)	52 (44–60)	70 (63–78)	1.94 (0–4)
Δ BL - day 14	57 (49–65)	58 (49–66)	35 (27–42)	77 (70–84)	1.82 (0–4)
**Combined marker early improvement plus plasma BDNF**					
Baseline	72 (65–79)	32 (24–40)	48 (40–56)	64 (56–72)	1.42 (0–3)
Δ BL - day 14	44 (36–52)	72 (65–79)	44 (36–52)	71 (64–79)	1.96 (0–4)
**Early Improvement baseline—day 14**					
	87 (82–93)	23 (16–30)	35 (27–43)	77 (70–84)	2.07 (0–4)

*95%-CI: 95% Confidence Interval; BDNF: brain derived neurotropic factor; BL, baseline; PPV, positive predictive value; NPV, negative predictive value*.

In the 199 patients with a severe MDD (HAMD_17_ ≥ 25), we found that significantly more patients with methylation at CpG-87 were remitter at endpoint (CpG-87: Chi^2^: 4.678, *df* = 3; *p* = 0.031, OR = 2.96) than patients without methylation (Figure 1B). Logistic regression analysis also revealed that the methylation status at BDNF promoter exon IV at site CpG-87 was significantly associated to remission of the depressive symptomatology (*R*^2^ = 4.5, ß = 0.866; *p* = 0.029). Additionally, patients with methylation at CpG-87 had a 2.96 higher likelihood to become remitter than patients without methylation, increasing the specificity from 42% in the entire group to 74% (Tables 2A,B).

#### P11 promoter methylation

The p11 promoter methylation levels did not differ between early improver and non-improver (*p* = 0.108) and remitter and non-remitter (*p* = 0.155). The logistic regression analyses also showed no association between the mean p11 promoter methylation and remission at endpoint (*p* = 0.055). The p11 methylation rate predicted later remission with high to very high sensitivity, but low to very low specificity. No differences were seen in the subgroup of severely depressed patients (data shown in Supplementary Table 2).

#### BDNF plasma levels

Mean (±SD) pBDNF level at baseline was 62 ± 886 pg/ml (range 0.4–3,952), there were no gender differences neither at baseline (*p* = 0.751) nor at week 2 (*p* = 0.656). BDNF plasma levels at baseline did neither differ between early improver and non-improver (*p* = 0.313) nor between remitter and non-remitter (*p* = 0.294). Mean change of pBDNF between BL and week 2 was −277 ± 828 pg/ml (range −5208 to 96) and between BL and week 8 −94.62 ± 745 pg/ml (range −3,330 to 3,119). Early improver and non-improver as well as remitter and non-remitter also did not differ in the change of pBDNF between baseline and day 14 (*p* = 0.117). Additionally, patients with or without methylation at CpG-87 did not differ in the change of pBDNF between BL and day 14 in the total group (methylated: −50.8 ± 840.6; unmethylated: −61.1 ± 907.1; *p* = 0.906) as well as in severely ill patients (methylated: 1.7 ± 798.3; unmethylated: −108.0 ± 829.7; *p* = 0.410). The number of patients with pBDNF increase was also not different in remitters and non-remitters (*p* = 0.922) (Figure 1C) as well as in improvers and non-improvers (*p* = 0.335). This was also true for the group of severely depressed patients (*p* = 0.534) (Figure 1D) for pBDNF increase).

The logistic regression analysis with the single markers pBDNF at baseline or the change of pBDNF from baseline to day 14 as predictors for remission as outcome showed no significant association between pBDNF and treatment outcomes. pBDNF at baseline or an early change of pBDNF from baseline to day 14 predicted remission with moderate sensitivity or specificity in all patients (Table 2A) and in severely depressed patients as well (Table 2B).

#### Early improver and non-improver and their relationship to later remission

**Figure 2:**
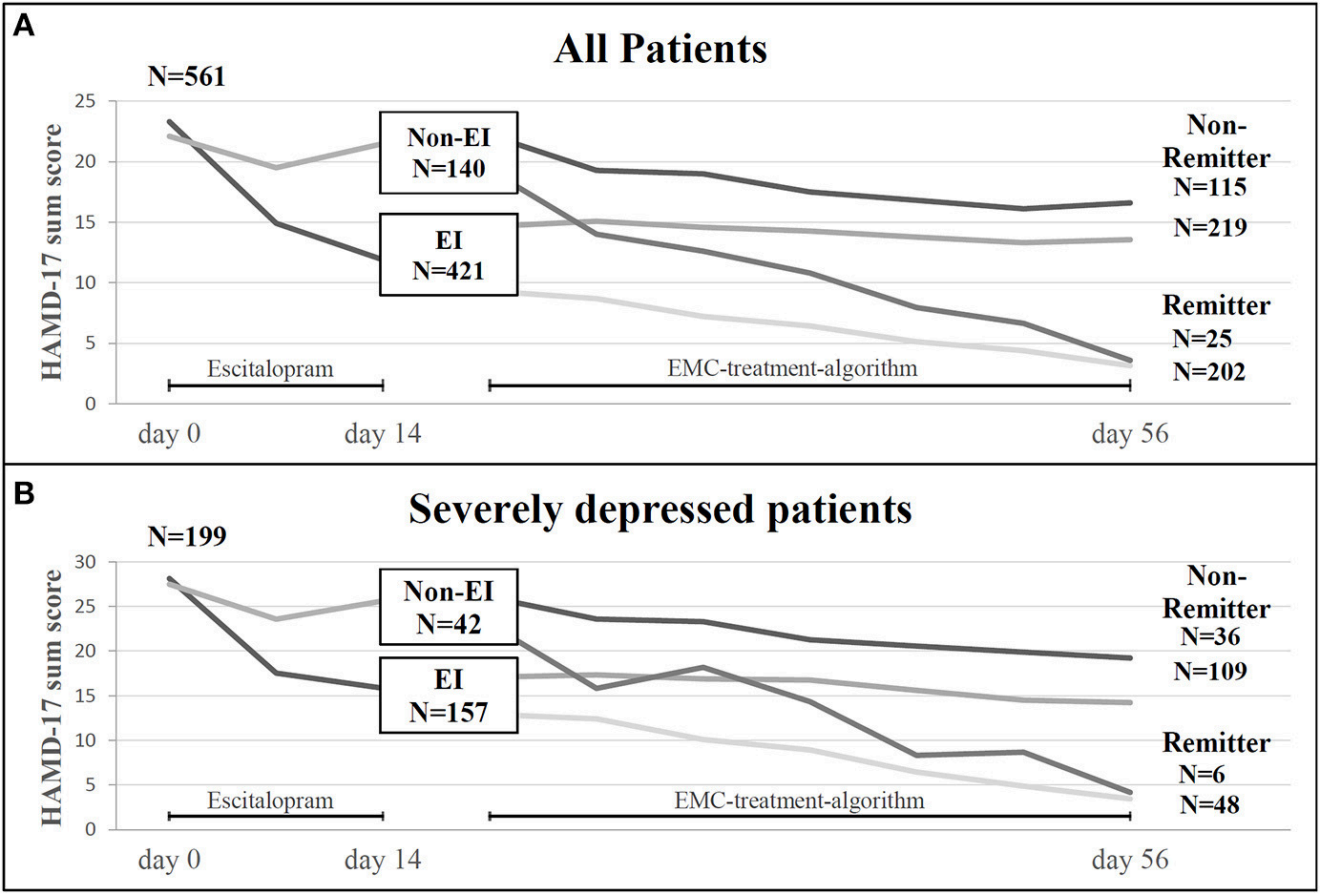
**(A)** Treatment courses in 561 patients with MDD included in the study (ITT sample): Number of patients experiencing an early improvement (EI) or early non-improvement (Non-EI) after 2 weeks of antidepressant treatment and number of patients with remission or non-remission after 8 weeks of treatment in relation to EI and Non-EI-status. **(B)** Treatment courses in 199 patients with severe MDD (HAMD-17 ≥25) included in the study: Number of patients experiencing an early improvement (EI) or early non-improvement (Non-EI) after 2 weeks of antidepressant treatment and number of patients with remission or non-remission after 8 weeks of treatment in relation to EI and Non-EI-status. EI, early improvement; EMC, early medication change; HAMD-17, Hamilton Depression rating Scale; N, number.

Four hundred and twenty-one (75%) of the 561 MDD patients showed an early improvement after 2 weeks of therapy compared to 140 patients showing no early improvement (Figure 2A). Two hundred and two (48%) patients with an early improvement were remitter at endpoint. However, 219 (52%) early improver were non-remitter at the end of the study. Of the 140 early non-improver, 25 (18%) were remitter whereas 115 (82%) became non-remitter at day 56. Early improver had a 4.24-fold higher likelihood to become remitter than early non-improver. As shown in Figure 2B, 79% (*n* = 157) of the 199 patients with a severe MDD were improver after 2 weeks of treatment. 30.6% (48 patients) of the improver became remitter at the end of the study, whereas 69.4% (109 patients) became non-remitter. Of the 42 non-improver of treatment (21%), 14.3% achieved remission at the end of the study and 85.7% (36 patients) were non-remitter. Among the severe depressed patients the early improvers had a 2.07-fold likelihood to become remitter.

### Combination of BDNF markers and early improvement to predict remission at endpoint

**Figure 3:**
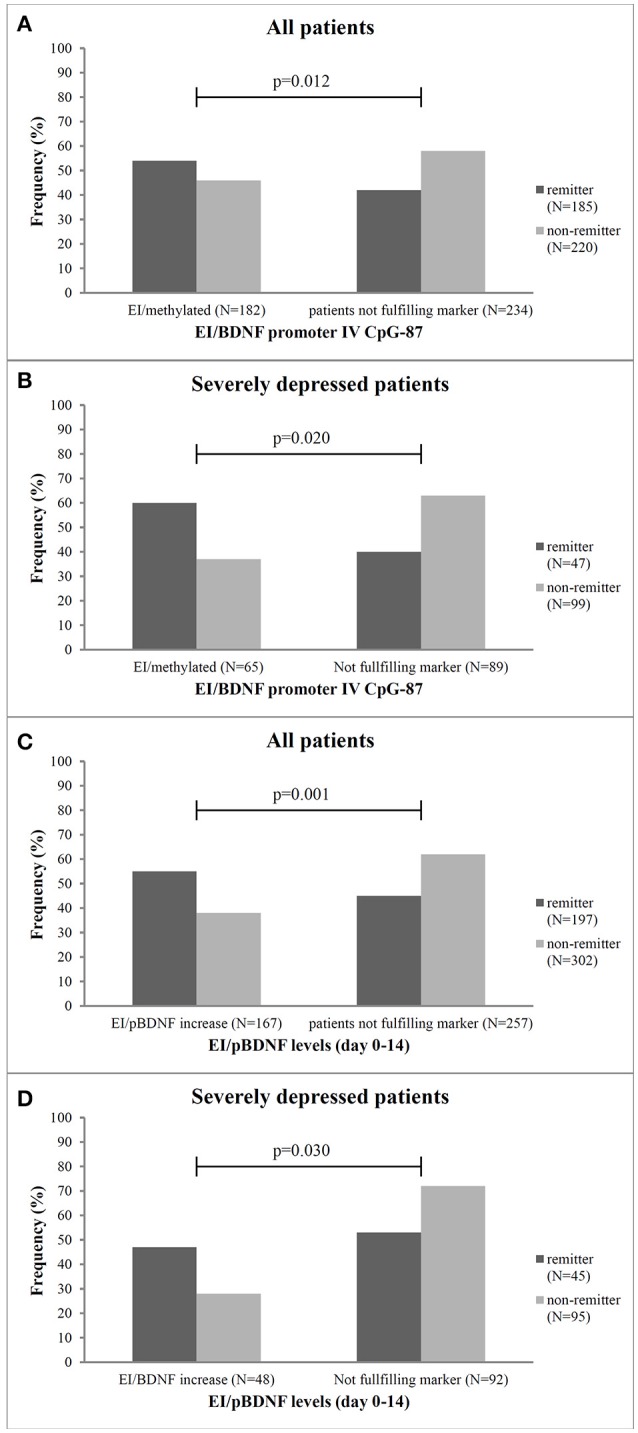
Frequency of remitter and non-remitter in relation to the combined marker of early improvement and methylation status at the CpG-87 site and pBDNF increase from baseline to week 2 for all patients (**A,C**, respectively) and the subgroup of severely depressed patients (**B,D**, respectively). EI, early improver; N, number; p, Chi^2^-test, patients not fulfilling marker, neither early improvement nor BDNF-methylation or pBDNF increase.

As shown in Table 2A, the combined marker of early improvement and methylation status at CpG-87 decreased the sensitivity (89–50), but increased the specificity of treatment prediction from 34 to 62%. The combined marker also led to an increase of Odd's ratios (BDNF exon IV promoter methylation as single marker OR: 0.92, combined with early improvement OR: 1.62). By combining early improvement with the methylation status at the other sites, sensitivity of prediction of remission was mostly high for the combined marker, but specificity was low, indicating that the combined marker at the other sites appears to be less predictive than the single marker “methylation status” (Table 2A). Patients with an early improvement and a methylated CpG-87 site at baseline more often became remitter (Chi^2^ = 22.6, *df* = 3, OR = 1.62; *p* = 0.001) after 8 weeks of antidepressant treatment as compared to all other patients (Figure 3A). Specificity of remission prediction was improved from 34 to 76% by the combined marker of pBDNF increase plus early improvement (OR: 3.78) (Table 2A). Patients with an early improvement and a pBDNF increase from baseline to day 14 more often became remitter at endpoint than patients without this marker (Chi^2^ = 43.1, *df* = 3, *p* = 0.001) (Figure 3C). All other combined markers showed no difference in the number of remitters.

Severely depressed patients with an early improvement and a methylated CpG-87 site at baseline more often became remitter (*p* = 0.02) after 8 weeks of antidepressant treatment as compared to the other severely depressed patients (Figure 3B). In these patients the combined marker of early improvement and methylation status at CpG-87 decreased the sensitivity (87–60%), but increased the specificity of treatment prediction from 23 to 63% (Chi^2^ = 6.9; *df* = 3; OR = 2.631; CI = 1.181–7.404; *p* = 0.031); resulting in a slightly lower OR as the single marker BDNF promoter methylation (OR: 2.96).

Specifity of remission prediction was improved from 23 to 72% by the combined marker of pBDNF increase plus early improvement (Chi^2^ = 8.9; *df* = 3; OR = 1.964; CI = 0.3175–4.213; *p* = 0.030) (Figure 3D and Table 2B).

## Discussion

Our results partially replicate and extent our previous findings regarding a role of BDNF exon IV promoter methylation for treatment response prediction in patients with MDD (11). We found in a group of patients with severe depression that BDNF exon IV CpG-87 methylation was associated with higher remission rates, meaning that patients with a methylation at BDNF exon IV CpG-87 more often became remitter than patients without a methylation at BDNF exon IV CpG-87. However, in the total group of patients methylation status at BDNF exon IV CpG-87 was not associated with remission at endpoint. In the total EMC study sample, BDNF exon IV CpG-87 hypomethylation was only associated with later non-remission if the biological marker was used in combination with the clinical marker early improvement.

Our data are not in line with our previous pilot study, in which we had found that all MDD patients—not only the severely depressed ones—who showed a hypomethylation at the CpG-87 site of the exon IV promoter region of the BDNF gene were less likely to benefit from the therapy with antidepressants (11). Although we do not know the cause of this difference, one possible explanation might be that in the current study only escitalopram or venlafaxine were study medications whereas in our pilot study all kinds of antidepressants without any restrictions were allowed.

In line with our findings, however, are results from one small study which examined the influence of 8 weeks of antidepressant treatment with citalopram on histone H3 lysine 27 trimethylation (H3K27me3) levels at promoter-IV of the BDNF gene and BDNF expression in severely depressed patients (49). This study showed increased BDNF mRNA levels in responders and significantly reduced H3K27me3 levels (a marker for silencing genes) at BDNF exon IV promoter, which showed a negative correlation with change in depression severity. In sum, this study and our findings support the assumption that epigenetic modifications play an important role for the therapeutic action of antidepressants and may even be a prerequisite for the onset of antidepressant action (9).

Contradictory to our findings are also other studies which found differences in the methylation status of the promoter in depressed individuals suffering from suicidal behaviors (50) and which showed that the response to classical antidepressant treatment could be predicted by classifying patients into “high-” and “low-methylated” individuals, i.e., individuals with low DNA methylation at BDNF P4 showed a greater reduction in suicidal ideation (51). A possible explanation for this difference might be the investigation of different regions of the BDNF promoter and the focus on depressed patients with and without suicidality.

It is unclear why we were only able to show the predictive role of BDNF exon IV promoter methylation for antidepressant response in the subgroup of severely depressed patients. Although depression severity is one important factor associated with the response to antidepressants, with patients with more severe depression showing a higher likelihood to become responders (37), it is unclear how depression severity interacts with epigenetic modifications of the BDNF promoter to modulate treatment response. One explanation for the different response prediction patterns between severely and less severely depressed patients could lie in the fact that more severely affected patients show less placebo response rates (52) and that drug effects in those patients are “true” drug effects based on neurobiological underpinnings as described here. Fitting to this hypothesis is the observation that non-pharmacological approaches to the treatment of MDD such as transcranial direct current stimulation (tDCS) did not find associations between BDNF and treatment response (53). Further studies should investigate differences in response prediction between severely and less severely affected patients, e.g., by adding other biomarkers (e.g., inflammatory markers), epigenetic modification of genes other than BDNF (e.g., MAOA-gene) or other epigenetic alterations of BDNF (histone methylation) which were not included in the current analyses.

Although this was not a mechanistic study, our data are in line with robust neurobiological findings, connecting the response to antidepressants to the capability of the drugs to increase BDNF expression. Monoaminergic drugs can increase BDNF expression, not only via the well-described pathway of cAMP response binding protein (54), activated via 30,50-cyclic adenosine monophosphate (cAMP), but also “via phosphorylation of methyl-CpG-binding protein 2 (MeCP2) which—in its unphosphorylated form—binds to the promoter and forms a repressor complex, but dissociates from the DNA upon phosphorylation” (55). Only if the promoter is methylated, the specific MeCP2 binding can occur, meaning that only in carriers of this methylation at the CpG site of the promoter this antidepressant-induced activation of BDNF can occur. This neurobiological mechanism may explain why severely depressed patients in our study with an unmethylated BDNF promoter site were unlikely to remit with continued treatment.

Early improvement, defined as a decrease of depression severity of ≥20% in the first 2 weeks of treatment, is one of the best investigated and most reliable predictors for response to antidepressant treatment (35, 36). The sensitivity (true positive rate) of early improvement on remission was high in our study (i.e., 89 out of 100 remitter were early improver at day 14; sensitivity: 89%), but the specificity (true negative rate) was low (i.e., only 34 out of 100 non-remitter showed a non-improvement at day 14 or in other words 66 out of 100 early improver became non-remitter after 8 weeks of treatment and were false positives). This result is in line with previous studies (22, 23) and highlights that further markers are needed in order to improve the specificity of response prediction. By combining early improvement with the methylation status at CpG-87, we found that the specificity of response prediction increased (from 34 to 62%), i.e., only 38 out of 100 patients with an early improvement and a methylation at CpG-87 site became non-remitter at the end of the study. Thus, false positives were significantly reduced by use of the combined marker (from 66 to 38 out of 100).

In severely depressed patients we found that the combined marker of early improvement and methylation status at CpG-87 only slightly decreased the prediction of later treatment outcome (decrease in sensitivity from 74 to 63%; decrease in sensitivity from 71 to 60%). This suggests that the single molecular marker seems to be particularly relevant/useful for response prediction in severely depressed patients and that for this group of patients the combined clinical and molecular marker has no advantage. These results need to be replicated and further studies should investigate possible neurobiological underpinnings of this finding before concrete conclusions can be drawn for the clinical significance of the results.

Our results are in line with our earlier findings in a rather small sample of patients with MDD (7, 33). In 39 patients with MDD, the combination of the early improvement signal with an increase in plasma or serum BDNF between baseline and day 7 increased the specificity of response prediction up to 100%. In the present study, the specificity of response prediction raised from 34 to 76% by combining the early improvement signal with an increase of BDNF between baseline and day 14, meaning that only 24 out of 100 patients with an early improvement and pBDNF increase between baseline and day 14 became non-remitter at the end of the study. Thus, false positives were significantly reduced by the use of the combined marker from 66 to 24 out of 100). In severely depressed patients, the combined marker of pBDNF increase and early improvement increased the specificity of treatment prediction to a similar extent. Our data are in contrast to a recently published study, which showed no evidence for a better prediction of response by a combined marker of pBDNF-increase and early improvement as compared to early improvement alone, which might be due to the very small sample size of 21 depressed patients (6).

Several limitations have to be kept in mind when interpreting the results of this study. First, the study is a secondary investigation and is not powered to this research question and did not use corrections for multiple comparisons. Therefore, our results should be interpreted carefully and should be verified in larger prospective samples. Second, we did not control for a possible influence of smoking behavior, as smoking has been shown to alter BDNF levels (56, 57). A third limitation is that blood samples for BDNF measurement were taken between 08:00 and 12:00 a.m. whereas a recent study showed that there are distinct fluctuations of pBDNF both in men and women over the day with an individual peak time unrelated to the clock time (58–60). A further limitation comprises the open delivery of treatment and the lack of a placebo control. Raters for the assessment of the efficacy outcomes, however, were blinded to group assignment and protocol medication.

The combination of clinical markers such as early improvement with molecular markers of the BDNF gene to predict treatment response is a new and innovative approach. If the predictive power of the combined markers can be replicated in further studies, this opens new avenues for the treatment of patients with MDD: A simple blood test at the initiation of antidepressant treatment and a test result within 2 weeks combined with the clinical marker of early improvement could guide physicians to change and/or optimize antidepressant therapy in patients who have a very low likelihood to respond. Further prospective and well-powered studies have to be designed to evaluate the efficacy of new treatment strategies in patients with such a very low likelihood of therapy response after 2 weeks of treatment.

## Data availability statement

The raw data supporting the conclusions of this manuscript will be made available by the authors, without undue reservation, to any qualified researcher.

## Author contributions

KL, ND, SW, AT, and HF: study concept and design. KL, ND, SW, KS, TF, AN, LM-E, SB, AT, and HF: acquisition, analysis, or interpretation of the data. KL, ND, SW, AT, and HF: drafting of the manuscript.

### Conflict of interest statement

KL, AT, HF, and SB are designated as inventors of the European patent number 12171541.1–2404 Method for predicting response or non-response to a mono-aminergic antidepressant. AT has received consultancy fees from Janssen and Novartis. The remaining authors declare that the research was conducted in the absence of any commercial or financial relationships that could be construed as a potential conflict of interest.
