# Supplementary material for: BDNF Plasma Levels and BDNF Exon IV Promoter Methylation as Predictors for Antidepressant Treatment Response
Source: Front Psychiatry. 2018 Oct 26;9:511. doi: 10.3389/fpsyt.2018.00511 (PMC6232909; doi:10.3389/fpsyt.2018.00511)
Supplement: Supplementary file 1 [file Table_1.DOCX]

Supplementary Material

BDNF Plasma Levels and BDNF Exon IV Promotor Methylation as Predictors for Treatment Response in Patients with Major Depressive Disorder

**Klaus Lieb, MD^1*#^, Nadine Dreimüller, MD^1#^, Stefanie Wagner, PhD^1^, Konrad Schlicht, MD^1^, Tanja Falter^2^, Alexandra Neyazi, MD³, Linda Müller-Engling³, Stefan Bleich, MD^3^, André Tadić, MD^1#^, Helge Frieling, MD^3#^**

*** Correspondence:** Klaus Lieb; klaus.lieb@unimedizin-mainz.de

# Supplementary Data

# Supplementary Figures and Tables

-

## Supplementary Figures

-

2. 2 Supplementary Table

Primer sets for amplification of BDNF and P11 promoter region.

| Oligo | Sequence | Fragment size | Genomic position |
| --- | --- | --- | --- |
| BDNF_fwd_1 | Ggg gga gga tta att gag tta gtt ttg | 277bp | NCBI NC_000011.9: 27723103  -27723380 |
| BDNF_fwd_2 | ttt gTt ggg gTt gga agt gaa aaT |  |  |
| BDNF_rev | ATA TAT ACT CCT TCT ATT CTA CAA CAA |  |  |
| BDNF_seqrev | aca aaa aaat ttc ata ct aa |  |  |
| P11_fwd_1 | TTGAGATAGAGTTTTATTTTG | 354bp | NC_000001.11: 151994447 - 151994801 |
| P11_fwd_2 | AGTTTTATTTTGTTATTTAGGTT |  |  |
| P11_rev | AAACCCTTTATTAAACAAAAAAT |  |  |
