# Supplementary material for: BDNF Plasma Levels and BDNF Exon IV Promoter Methylation as Predictors for Antidepressant Treatment Response
Source: Front Psychiatry. 2018 Oct 26;9:511. doi: 10.3389/fpsyt.2018.00511 (PMC6232909; doi:10.3389/fpsyt.2018.00511)
Supplement: Supplementary file 2 [file Table_2.DOC]

Supplementary table 2: Sensitivity and specificity of early improvement and its combination with P11 for prediction remission in patients with MDD

|  | **Sensitivity**  **(95%-CI)** | **Specificity (95%-CI)** | **PPV**  **(95%-CI)** | **NPV**  **(95%-CI)** | **Odd´s ratio**  **(95%-CI)** |
| --- | --- | --- | --- | --- | --- |
| **Outcome: remission at endpoint** | | | | | |
| P11 38  P11 44  P11 78  P11 112  P11 114  P11 128  P11 207  P11 211  P11 216  P11 244  P11 254  P11 256  P11 260  P11 314  P11 mean | 81 (78-84)  97 (95-98)  91 (88-93)  0.4 (0-1)  86 (83-89)  95 (93-97)  88 (85-91)  92 (89-94)  1 (0-2)  0.3 (0-1)  83 (80-86)  86 (83-89)  1 (0-2)  14 (11-17)  90 (87-92) | 19 (16-23)  1 (0-2)  11 (9-14)  96 (94-98)  18 (15-21)  1 (0-2)  17 (14-20)  12 (9-15)  91 (88-93)  97 (95-98)  20 (17-24)  15 (12-18)  96 (94-98)  90 (87-92)  15 (12-18) | 46 (42-50)  48 (44-52)  47 (43-51)  47 (43-51)  48 (44-52)  47 (43-51)  48 (44-52)  48 (44-52)  50 (46-54)  83 (80-86)  48 (44-52)  48 (44-52)  61 (57-65)  56 (52-60)  49 (45-53) | 54 (50-58)  71 (67-75)  56 (52-60)  53 (49-57)  59 (55-63)  58 (54-62)  63 (59-67)  62 (58-66)  53 (49-57)  54 (50-58)  58 (54-62)  55 (51-59)  53 (49-57)  54 (50-58)  63 (59-67) | 0.97 (0-2)  2.31 (1-4)  1.15 (0-2)  1.02 (0-2)  1.33 (1-3)  1.24 (1-3)  1.54 (1-3)  1.49 (1-3)  1.11 (0-2)  1.49 (1-3)  1.25 (1-3)  1.13 (0-2)  1.81 (1-3)  1.47 (1-3)  1.59 (1-3) |
| *Combined marker early Improvement plus P11* | | | | | |
| P11 38  P11 78  P11 114  P11 207  P11 254  P11 256  P11 mean | 72 (68-76)  53 (49-57)  75 (71-79)  79 (75-82)  70 (66-74)  76 (72-80)  79 (75-82) | 44 (40-48)  55 (51-59)  44 (40-48)  20 (17-24)  49 (45-53)  41 (37-45)  41 (37-45) | 52 (48-56)  18 (15-21)  55 (51-59)  55 (51-59)  55 (51-59)  53 (49-57)  55 (51-59) | 65 (61-69)  86 (83-89)  67 (63-71)  43 (39-47)  65 (61-69)  65 (61-69)  69 (65 – 73) | 1.98 (1-4)  1.37 (1-3)  2.40 (1-4)  0.92 (0-2)  2.25 (1-4)  2.14 (1-4)  2.68 (2-4) |

Legend to supplementary table 2: 95%-CI: 95% Confidence Interval; PPV: positive predictive value; NPV: negative predictive value; BDNF: brain derived neurotropic factor; BL: baseline
